# Punching Shear Strength of FRP-Reinforced Concrete Slabs without Shear Reinforcements: A Reliability Assessment

**DOI:** 10.3390/polym14091743

**Published:** 2022-04-25

**Authors:** Soliman Alkhatib, Ahmed Deifalla

**Affiliations:** 1 Basic Sciences Department, Faculty of Engineering and Technology, Future University in Egypt, New Cairo 11835, Egypt; soliman.alkhatib@fue.edu.eg; 2 Structural Engineering and Construction Management Department, Faculty of Engineering and Technology, Future University in Egypt, New Cairo 11835, Egypt

**Keywords:** punching shear, slabs, GFRP, CFRP, FRP, reliability

## Abstract

The recent failure of buildings because of punching shear has alerted researchers to assess the reliability of the punching shear design models. However, most of the current research studies focus on model uncertainty compared to experimentally measured strength, while very limited studies consider the variability of the basic variables included in the model and the experimental measurements. This paper discusses the reliability of FRP-reinforced concrete slabs’ existing punching shear models. First, more than 180 specimens were gathered. Second, available design codes and simplified models were selected and used in the calculation. Third, several reliability methods were conducted; therefore, three methods were implemented, including the mean-value first-order second moment (MVFOSM) method, the first-order second moment (FOSM) method, and the second-order reliability method (SORM). A comparison between the three methods showed that the reliability index calculated using the FOSM is quite similar to that using SORM. However, FOSM is simpler than SORM. Finally, the reliability and sensitivity of the existing strength models were assessed. At the same design point, the reliability index varied significantly. For example, the most reliable was the JSCE, with a reliability index value of 4.78, while the Elgendy-a was the least reliable, with a reliability index of 1.03. The model accuracy is the most significant parameter compared to other parameters, where the sensitivity factor varied between 67% and 80%. On the other hand, the column dimension and flexure reinforcement are the least significant parameters compared to other parameters where the sensitivity factor was 0.4% and 0.3%, respectively.

## 1. Introduction

Vital infrastructures suffer from the risk of brittle collapse because of a lack of maintenance, especially under severe environmental conditions causing steel corrosion. Thus, fiber-reinforced polymers (FRP) are replacing steel because of their excellent properties, avoiding problems caused by steel corrosion [1,2,3,4,5,6,7,8,9,10]. FRP usage in structural engineering applications started in the area of strengthening various elements [11,12,13,14,15], then FRP-reinforced concrete [16] and later fiber-reinforced concrete [17,18,19]. It is worth noting that FRP types have significantly different mechanical properties, corrosion, and fatigue/creep resistance. For example, glass FRP (FRP) has lower tensile strength and higher elongation at break. However, it is easy to degrade in a concrete alkaline environment because an Si-O- skeleton in glass fiber reacts with hydroxyl ions in an alkali environment. In addition, the fatigue and creep resistances of GFRP are relatively poor during long-term service. In contrast, carbon FRP (CFRP) has higher strength, corrosion, and fatigue resistance. However, its lower elongation at break easily leads to the brittle fracture failure of reinforced infrastructures [20,21,22]. However, FRP-reinforced concrete slabs are spreading worldwide because of their superior properties [7,23,24]. In addition, several design codes and guidelines are being developed that include provisions for the FRP-reinforced concrete slabs [7,23,24]. Moreover, implementing artificial intelligence is becoming popular in providing reliable methods for the shear and punching shear of beams and slabs [25,26,27]. Most of the existing design methods for slab–column connections lack the physical sense, which is due to being empirical or semi-empirical [5]. Thus, advanced methods for assessing the reliability of design models are a mandate [28,29,30,31,32].

Well-designed buildings can suffer from catastrophic punching shear failure because of variability associated with design models: aleatory and epistemic. The aleatory variability is due to the uncertainty of basic variables affecting the punching shear problem. In comparison, the epistemic variabilities are due to uncertainty in the model caused by unconsidered variables and a lack of accuracy in the model. Thus, to assess a design model while considering both types of variability, state-of-the-art reliability analysis methods are required. This analysis will provide a more precise evaluation of these design models’ safety level and precision. Uncertainty quantification—a family of recent techniques proposed by the mathematical community—seems a promising tool that significantly enhances the structure’s safety analysis. In particular, stochastic collocation and Galerkin methods [33,34], moment equations [35,36], and the perturbation method [35,37,38,39] are efficient and powerful approaches that outperform the Monte Carlo ones. For example, the mean-value first-order second moment (MVFOSM) method, the first-order second moment (FOSM) method, and the second-order reliability method (SORM) are being used for reliability in structural analysis and design [28,31,40,41,42,43].

Furthermore, FOSM and SORM are the most dependable structural reliability methods. Both methods are analytical approximations in which the reliability index is interpreted as the shortest distance from the origin to the performance function surface in a standardized normal space (Z-space) and (design point), which is found using an iterative method [44]. When the performance function is strongly nonlinear, accuracy issues arise because a linear function approximates it in Z-space at the design point [45,46]. The second-order reliability method (SORM) was developed to improve the accuracy of FOSM. SORM is obtained by approximating the performance function surface in Z-space at the design point with a second-order surface. However, some authors still believe that FOSM is an efficient method when the performance function is linear or nearly linear and that it produces accurate results [47,48].

This paper conducted a reliability assessment of the state-of-the-art strength models. First, an extensive experimental database of more than 180 FRP-reinforced concrete slabs under punching shear was collected. Second, selected design models were outlined. Moreover, the MVFOSM, the FOSM, and the SORM were implemented and compared with each other. Third, reliability indices (β) were used for evaluating the safety level and thus the failure probability. Finally, concluding remarks regarding the accuracy of various reliability analyses and available strength models from the literature were outlined and discussed.

## 2. Experimental Database and Selected Models

**Figure 1:**
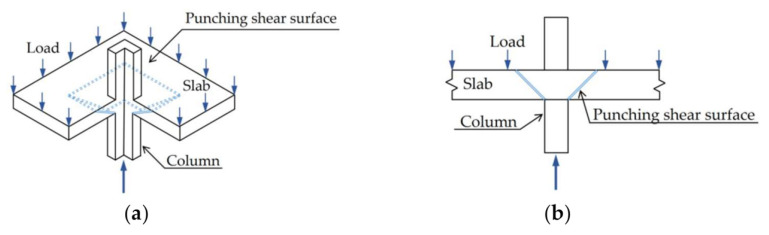
Punching shear failure, (**a**) Stereogram, and (**b**) Profile. Reprinted from ref. [49].

**Figure 2:**
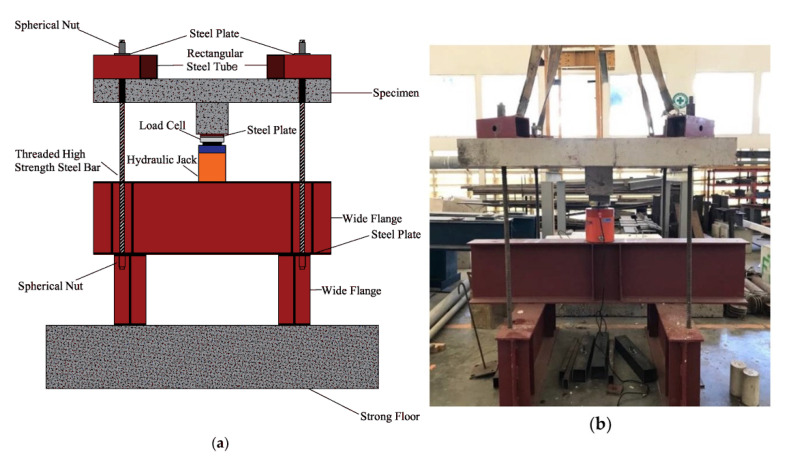
Punching shear loading test setup, (**a**) schematic; (**b**) actual. Reprinted from ref. [50].

**Figure 3:**
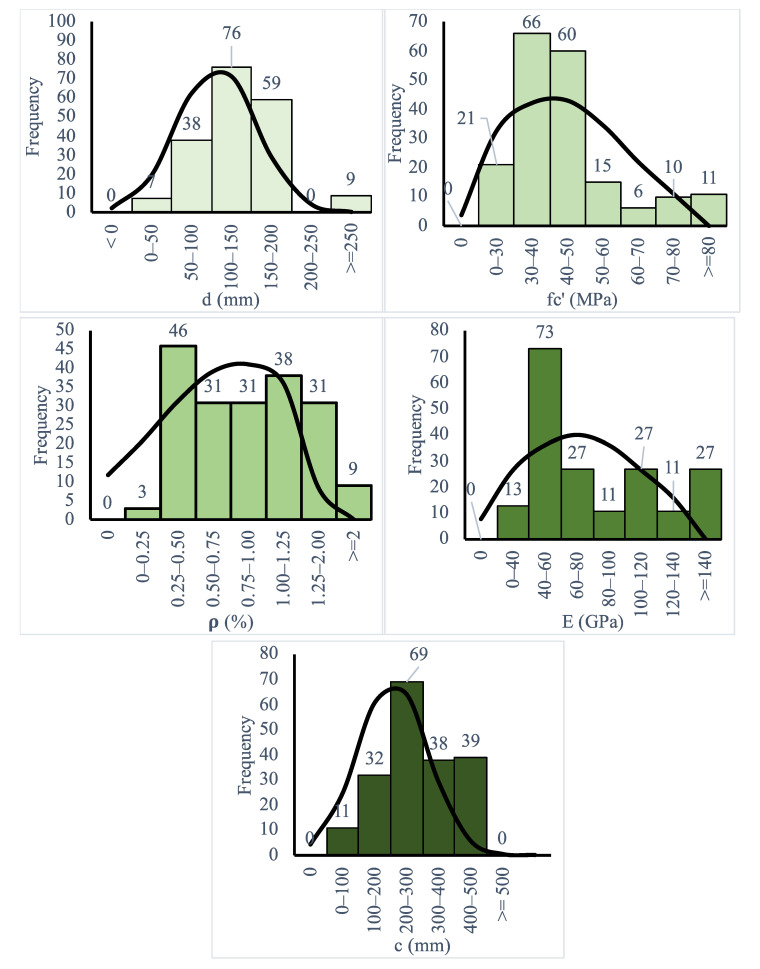
Experimental database description.

Although FRP reinforcement has different diameters, to consider the influence of the material, both young’s modulus and the flexure reinforcement ratio were implemented. It is worth noting that all gathered slabs were subjected to punching shear loading, as shown in Figure 1 [49]. All slabs failed suddenly under punching shear, as shown in Figure 2 [50]. Table 1 and Figure 3 show the description of an extensive experimental database used in the calculation of the selected model variability, where Young’s modulus (E), effective depth (d), concrete compressive strength ($f_{c}^{\prime }$), flexure reinforcement ratio (ρ), column dimensions (b), and slab dimensions (A, B) are listed. The description of the experimental database is shown in Table 1, and details are listed in another research study [5].

Over the last four decades, several design models for FRP-reinforced concrete slabs under punching shear have been available. Table 2 shows selected models, which are design codes, design guidelines, and models developed for the case of FRP-reinforced concrete slabs under punching shear. Models were selected based on simplicity and suitability for design or recently developed. Since these models were developed using an experimental database (i.e., semi-empirical or empirical), their reliability needs to be examined. In addition, identifying the effect of basic variables on the reliability of such design codes and models is highly demanded.

## 3. Uncertainties of the Selected Models and Basic Variables

### 3.1. Basic Variables

Uncertainties in structural engineering design vary from being obvious to those rarely recognized by practitioners. When the strength calculated using available models is compared with that measured experimentally, there is variation that is due to uncertainties in the model assumptions and measurements from the test results. Thus, the actual capacity is calculated in such a way that:

(1) $$R_{d}=\theta R_{dmodel}$$

where $R_{dmodel}$ is the capacity calculated using the selected models shown in Table 2, $R_{d}$ is the measured capacity, and θ is a parameter representing the model variability. The θ is calculated as the ratio of measured capacity to calculated capacity using the selected model. Table 3 shows the statistical measures for the state of the art collected and used to calculate the model uncertainty. The statistical values obtained from normal distribution need to be corrected because the capacity is believed to be log-normal distribution and the test results variability [89]. The average ($\mu_{\theta }$) of θ is calculated such that:

(2) $$\mu_{\theta }=e^{\mu +0.5\sigma^{2}}$$

where μ is the average based on normal distribution and $\sigma^{2}$ is the standard deviation based on a normal distribution, while the coefficient of variation $\left(CoV_{\theta }\right)\;$is such that:

(3) $$CoV_{\theta }=\sqrt{CoV_{LN,\;conv}{}^{2}+CoV_{tests}{}^{2}}=\sqrt{e^{\sigma^{2}}-1-0.05^{2}},$$

where $CoV_{LN,\;conv}$ is the coefficient of the variation based on log-normal distribution and $CoV_{tests}$ is the coefficient of the variation for test results taken as 0.05.

### 3.2. Basic Variables

Table 4 shows the statistical measured based on the JCSS probabilistic model code [90] for the various basic variables considered by the selected models.

## 4. Reliability Analysis Methods

Reliability is the probability that the random variables $U≔(U_{1},U_{2},U_{3},U_{4},\ldots ,U_{n}$*)* are in the safe region where $G\left(U\right)>0$. The probability of failure is defined as the probability *P*{$G\left(U\right)$ < 0}. Alternatively, it is the probability that the random variables U_n_ are in the failure region defined by *G*(*U*) < 0. If the joint probability density function (pdf) of *U* is$\;F_{U}\left(U\right)$, the likelihood of failure is evaluated with the integral

(4) $$\;P_{F}=P\left\{G\left(U\right)<0\right\}=\int F_{U}\left(U\right)\;dU$$

and the likelihood of reliability is calculated as:

(5) $$R=1-P_{F}=P\left\{G\left(U\right)>0\right\}$$

Calculating the integral in this equation is typically difficult because of various factors such as the complexity of the ultimate function and the number of random variables, and numerical calculations are not always preferable in most cases. To avoid evaluating such integrals, employ probability and statistics approximation methods. The FOSM, SORM, and the Mont-Carlo simulation with importance sampling (MC-IS) are all very efficient methods for calculating failure probability or a reliability index. These methods’ basic idea is to simplify computation by simplifying the integrand $F_{U}\left(U\right)$. Moreover, approximating the performance function *G*(*U*), a solution to this equation will be easily obtained through simplification and approximation. The validation of the inequality equation is at the heart of structure reliability.

**Figure 4:**
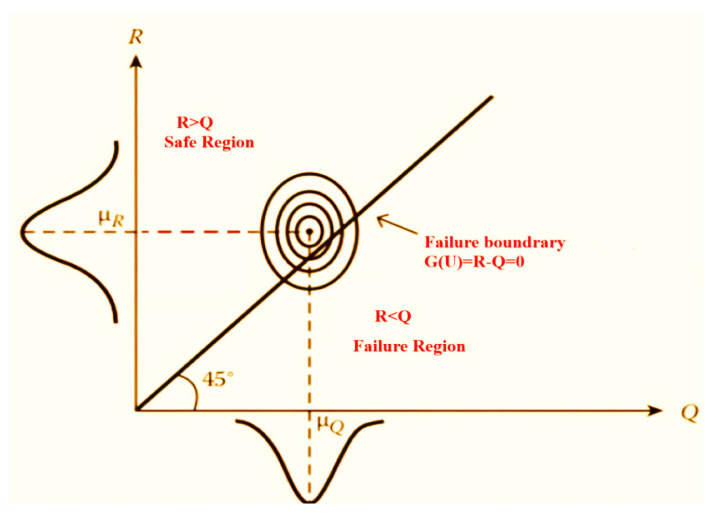
Theory of reliability and conditions of failure.

(6) R≥Q

where *R*, in general, presents the resistance and *Q* presents the load. Figure 4 shows the safe and failure regions when *R* and *Q* are normally distributed. When it is equal, which is the most critical situation, it can be written in a simpler form as follows:

(7) $$G\left(U\right)=R-Q=0$$

**Figure 5:**
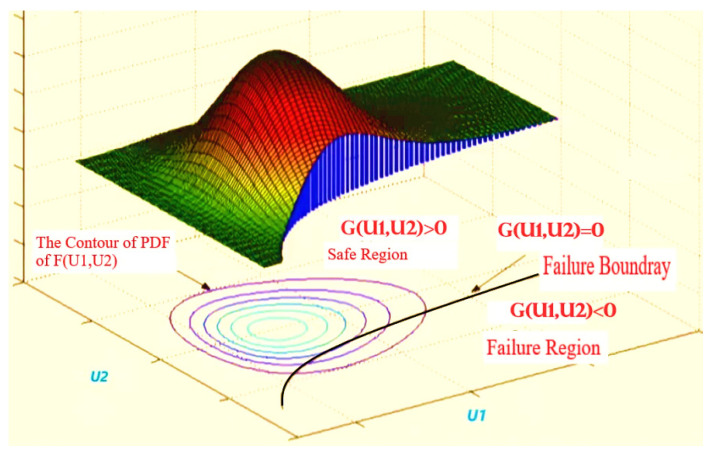
The joint PDF of *U*_1_ and *U*_2_ and safe region and failure region of the performance function of two random variables.

All the random variables (*U*) in this section are assumed to be mutually independent. In the case of two random variables$\;U_{1}$ and $U_{2}$, the joint probability density function (pdf) and its contours, which are projections of the surface of the $(U_{1}$, $U_{2})$ plane are illustrated in Figure 5. All the contour points have similar probability densities or have the same values of $F_{U}\left(U\right)$. On the $(U_{1}$, $U_{2})$ plane, the integration boundary *G*(*U*) = 0 is also plotted. The most common reliability analysis methods are used in this paper, including the first-order second moment method, which involves three cases: the first is the mean-value first-order second moment (MVFOSM) method, which uses the mean and standard deviation only to calculate the reliability without regard to the type of the distributions. It usually gives inaccurate results, especially when the performance function is nonlinear. The second is the Hasofer–Lindt matrix procedure (HL-MP), when the random variables are normally distributed. The third is the Rackwitez–Fissler improved matrix procedure (RF-IMP) when some are non-normally distributed. Finally, the second-order reliability method (SORM) was used to validate the first accuracy of the order method, which was created to address the problem of high nonlinearity in the performance function of many random variables.

### 4.1. The Mean-Value First-Order Second Moment (MVFOSM) Method

**Figure 6:**
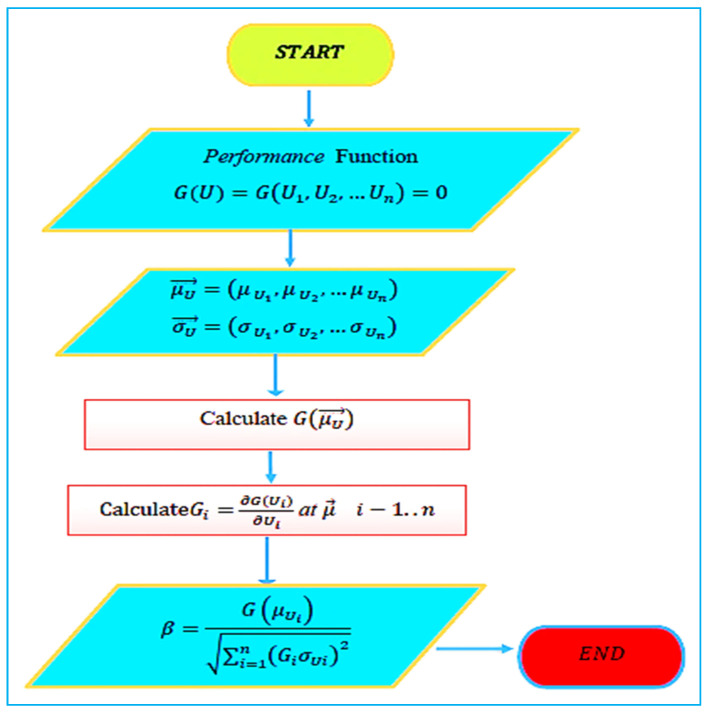
Mean-value first-order second moment procedure.

The mean-value first-order second moment (MVFOSM) method involves the linear approximation of a given performance function and its derivatives to a first-order Taylor series at the mean values of random input variables: $\;U^{*}={\left(U_{1\;}^{*},\;U_{2\;}^{*},\;\ldots ,\;U_{n\;}^{*}\;\right)}^{T}$. The procedure of the MVFOSM is shown in Figure 6. The first order of the Taylor series of the performance function is such that:

(8) $$G\left(U\right)=G\left(\;U^{*}\right)+\nabla G\left(\;U^{*}\right)\left(U-U^{*}\right)$$

while

(9) $$\nabla G\left(\;U^{*}\right)=({\left.\frac{\partial G}{\partial U_{i}}\right|}_{U^{*}})$$

The expected value of $\mu_{X}$ is given by:

(10) $$\mu_{U}=\;G\;\left(U^{*}\right)$$

The variance $\sigma_{U}^{\;2}=\mathrm{Var}\;\left(U\right)$ is given by:

(11) $$\sigma_{U}^{\;2}=\overset{n}{\underset{i=1}{\sum }}{\left(\nabla G\left(\;U^{*}\right)\right)}^{2}\;\sigma_{U_{i}}^{\;2}\;$$

So, the reliability index β is determined by:

(12) $$\beta =\frac{\mu_{U}}{\sigma_{U}}\;$$

The probability of failure $P_{f}$ can be determined as:

(13) $$P_{f}=\Phi \;\left(-\beta \right)=1-\Phi \;\left(\beta \right)$$

where Φ is the integral of the standard normal distribution; the disadvantage of this technique is that it may result in *β*-resolution. This inaccuracy is due to the linear implementation. As a result, this technique cannot adequately represent nonlinear or significant differences. Therefore, improving this method using the FOSM, SORM, or the Monte Carlo simulation to produce a more accurate *β*.

### 4.2. First Order Second Moment (FOSM)

The first-order second moment (FOSM) technique was introduced to avoid the struggles of the first-order approximation for the Taylor expansion implemented by the MVFOSM. The main point of this method is to expand the performance function as a Taylor expansion at the design point and not at the mean’s vector and then make a certain iterative linear approximation to find the accuracy of the reliability index. The linearization of the design function is implemented at the design-point U*, which is the most probable failure point. In addition, it requires less computation time. Therefore, the design-point U* allocation governs the technique’s efficiency. Several techniques within the FOSM will be discussed and implemented in this section.

#### 4.2.1. Hasofer and Lindt Matrix Procedure (HL-MP)

Hasofer and Lindt [42,43] propose two alternative procedures to implement FOSM, the simultaneous technique equation procedure (HL-STE) and the matrix procedure (HL-MP). These two procedures have a more efficient effect than the MVFOSM when the parameter distributions are normally distributed. The (HL-MP) is favored because of the many random parameters of the set function. While using the (HL-STE) and (HL-MP), detailed information on the type of distribution for each random parameter is not needed as it is assumed to be normally distributed. When some random variables are non-normally distributed, (HL-MP) and (HL-STE) result in an inaccurate relation between the *β* and the performance function. Thus, an improvement is needed to calculate an accurate *β*, which Rackwitez–Fissler can do. The improved matrix procedure (RF-IMP) will be introduced to deal with normal and non-normal distributions.

#### 4.2.2. Rackwitez–Fissler Improved Matrix Procedure (RF-IMP)

**Figure 7:**
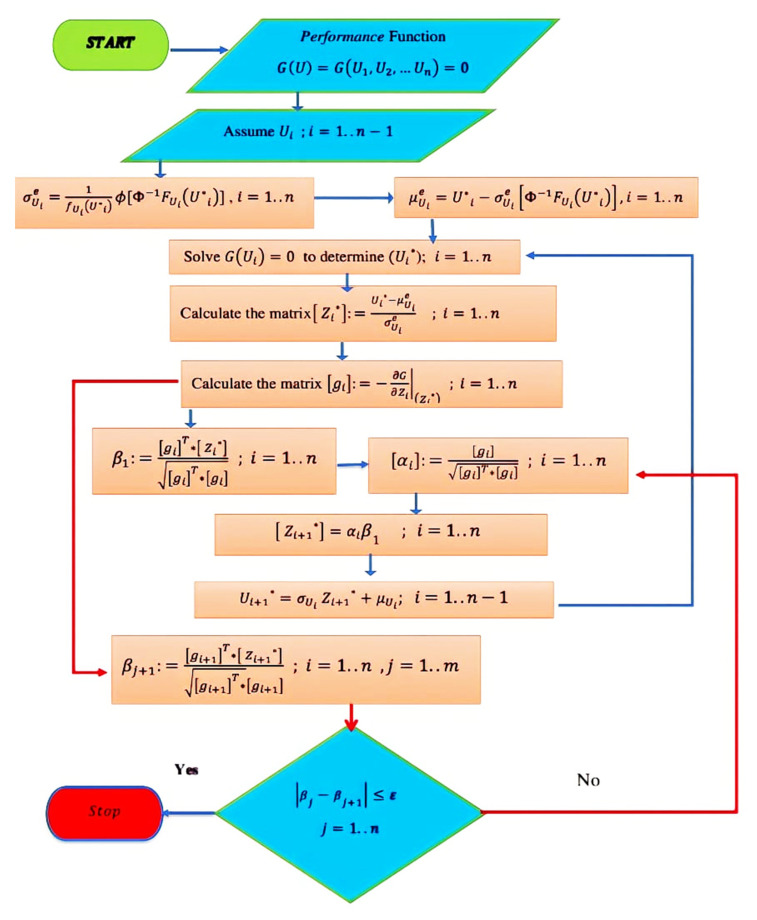
Procedure for calculating reliability index using the FOSM (HL-MP and RF-IMP).

The RF-IMP technique’s basic idea is to use an equivalent value for the mean and standard deviation for each non-normally distributed parameter. Figure 7 shows the flow chart of the procedure of (RF-IMP) and can be used for (HL-MP) if all random variables are normally distributed. Suppose that $F_{U}\left(U\right)$ is the cumulative distribution function (CDF) and $f_{U}\left(U\right)$ is the probability density function (PDF). Mathematically, calculating an equivalent mean $\mu_{U}^{e}$ and an equivalent standard deviation $\sigma_{U}^{e}$ such that:

(14) $$\mu_{U}^{e}=U^{*}-\sigma_{U}^{e}\left[\Phi^{-1}F_{U}\left(U^{*}\right)\right]$$

(15) $$\sigma_{U}^{e}=\frac{1}{f_{U}\left(U^{*}\right)}\phi \left(\frac{U^{*}-\mu_{U}^{e}}{\sigma_{U}^{e}}\right)=\frac{1}{f_{U}\left(U^{*}\right)}\phi \left[\Phi^{-1}F_{U}\left(U^{*}\right)\right]$$

where Φ and ϕ are CDF and PDF for the standard normal distribution. For each of the non-normal distribution values in the design vector $U^{*}$, an equivalent $\mu_{U}$ and $\sigma_{U}$ is calculated. For the normally distributed values of the vector $U^{*}$, no equivalent values are needed where the actual values of the parameters are adequate. For example, an equivalent normal parameter for a log-normal random parameter U can be calculated such that:

(16) $$\sigma_{U}^{e}=U^{*}\sigma_{lnU}$$

(17) $$\mu_{U}^{e}=U^{*}\left[1-\ln\left(U^{*}\right)+\mu_{lnU}\right]$$

where $U^{*}$ is the design point value and $\sigma_{lnU}$ and $\mu_{lnU}$ are the distribution parameters for the log-normal distribution. Hence, the calculation of reliability in this paper needs to use the RF-IMP to find the equivalent average and standard deviation $\theta_{R}$ and $f_{c}^{\prime }$ The procedure of this method is as follows:

- Starting with determining the limit state function $G\left(U_{i}\right)=0$, *i* = 1 … *n*.
- Assume *n* − 1 value for vector $U_{i}$.
- Solve the *G*$\left(U_{i}\right)=0$ for the remaining random parameter to find the design vector ($U^{*}$).
- For each non-normal distribution, determine the equivalent mean and standard deviation using $\mu_{U}^{e}=U^{*}-\sigma_{U}^{e}\left[\Phi^{-1}F_{U}\left(U^{*}\right)\right]$, $\sigma_{U}^{e}=\frac{1}{f_{U}\left(U^{*}\right)}\phi \left(\frac{U^{*}-\mu_{U}^{e}}{\sigma_{U}^{e}}\right)=\frac{1}{f_{U}\left(U^{*}\right)}\phi \left[\Phi^{-1}F_{U}\left(U^{*}\right)\right]$.
- Determine the reduced vector $Z^{*}$ where $Z_{i}{}^{*}=\frac{U_{i}{}^{*}-\mu_{U}^{e}{}_{i}}{\sigma_{U}^{e}}$ for each parameter of the design vector ($U^{*}$).
- Differentiate partially *G*(Z) with respect to vector$\;Z^{*}$, $\nabla G\left(\;Z^{*}\right)=-{\left.\frac{\partial G}{\partial Z_{i}}\right|}_{\left(Z_{i}{}^{*}\right)}\;;\;i=1,2\ldots n$.
- Calculate an estimate of the index reliability $\beta =\frac{{\left[\nabla G\left(\;Z^{*}\right)\right]}^{T}*Z^{*}}{\left\|\nabla G\left(\;Z^{*}\right)\right\|}$, where $Z^{*}={\left[Z_{1}^{*}\ldots Z_{n}^{*}\right]}^{T}$.
- Calculate the sensitivity factor $\alpha =\frac{{\left[\nabla G\left(\;Z^{*}\right)\right]}^{T}}{\left\|\nabla G\left(\;Z^{*}\right)\right\|}$.
- Using the formula $Z^{*}{}_{i}=\alpha_{i}\beta$, find the new design vector for the *n* − 1 of the parameter.
- Determine the corresponding design vector in the original coordinate, where $U_{i}{}^{*}=\sigma_{U_{i}}Z_{i}{}^{*}+\mu_{U_{i}}$.
- Solve Function *G*(U)=0 to determine the value of the remaining random parameter.
- Repeat steps 5 to 10 until β converges.

Because the (RF-IMP) method employs the first order of the Taylor series, it provides adequate values of the reliability index when the performance function has only one minimum distance point, and the function is nearly linear close to the design point. However, when the performance function is highly nonlinear, or the number of random variables is extremely large, the reliability index value may give an unreliable and inaccurate result. To avoid this problem and improve the (RF-IMP), the hypersurface’s curvature that presents the performance function is calculated. The curvature of any equation is related to the second-order derivatives with respect to the basic variables at the considered point. For our study, the Taylors series second order will be implemented at the design point obtained from the FOSM. This SORM will be discussed in the next section.

### 4.3. The Second-Order Reliability Method (SORM)

**Figure 8:**
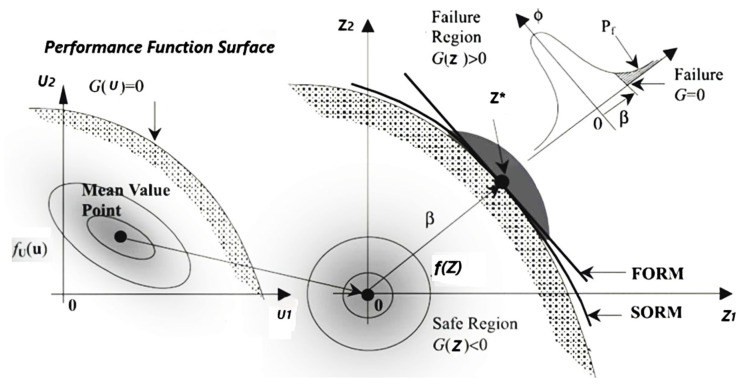
FSOM (RF-IMP) and the SORM method.

The second-order reliability method (SORM) was introduced as an improvement for the FOSM (RF-IM), where additional information about the curvature of the performance function is included [42]. SORM relies mainly on three parameters: the curvature radius at the design point, the number of random variables, and the first-order reliability index. More detail on this method can be found in [42,43]. The Taylor expansion to approximate the performance function at the design point is given by:

(18) $$G\left(U\right)=G\left(\;U^{*}\right)+\nabla G\left(\;U^{*}\right){\left(U-U^{*}\right)}^{T}+\frac{1}{2}\left(U-U^{*}\right)[\nabla^{2}G\left(\;U^{*}\right){\left(U-U^{*}\right)}^{T}]$$

where $[\nabla^{2}G\left(\;U^{*}\right)]={\left.\left[\frac{\partial 2G}{\partial U_{i}\partial U_{j}}\right]\right|}_{U^{*}}i=1‥n,\;j=1‥n$ is the Hessian matrix of $G\left(U\right)\;\mathrm{at}\;U^{*}$. The SORM is initially determined by the (FOSM-RF-IMP) results for the reliability index, the final design point, and the directional cosine vector at this final design point. The SORM’s primary goal is to improve the accuracy of the reliability index value calculated by the first-order reliability method (RF-IMP). This methodology begins by determining the principal curvature of the hypersurface. Depending on the nonlinearity of the performance function and the number of variables, the curvature calculation can produce positive, negative, or complex values. Various nonlinear approximate methods have been proposed [40,42,43]. The present paper uses Breitung’s SORM method, which uses a parabolic approximation; that is, it does not use a general second-order approximation. It uses the theory of asymptotic approximation to derive the probability estimate. The asymptotic formula is accurate only for large values of β. However, if the value of β is low, the SORM estimate could be inaccurate. Figure 8 shows the difference between FOSM and SORM [91], which explains why the curvature of the hypersurface must be calculated to provide an accurate value for reliability. Suppose the number of variables is not large enough, or the case of a very large curvature radius. In that case, the reliability index value between RF-IMP and SORM is quietly identic.

The procedure of this method starts by calculating the reliability index, the directional cosine vector *α* = $\left[\alpha_{i}\right]$, and the final design point in the original variables U* using the FOSM-based method (RF-IMP). Then, depending on the type of distribution, convert all variables from their original to standard form (when a random variable is non-normal, using the equivalent normal mean and equivalent standard deviation). Finally, the second-order estimate of the probability of failure can be computed using Breitung’s formula:

(19) $$P_{f_{2}}=\Phi \left(-\beta \right)\overset{n-1}{\underset{i=1}{\prod }}{\left[1+\beta \kappa_{i}\right]}^{-\frac{1}{2}}\;$$

where $\kappa_{i}$ is the principal curvature of the failure surface at the design point.

Finally, the SORM reliability index $\beta_{\mathrm{SORM}}$ is computed in such a way that:

(20) $$\beta_{\mathrm{SORM}}=-\Phi^{-1}\left(P_{f_{2}}\right).$$

**Figure 9:**
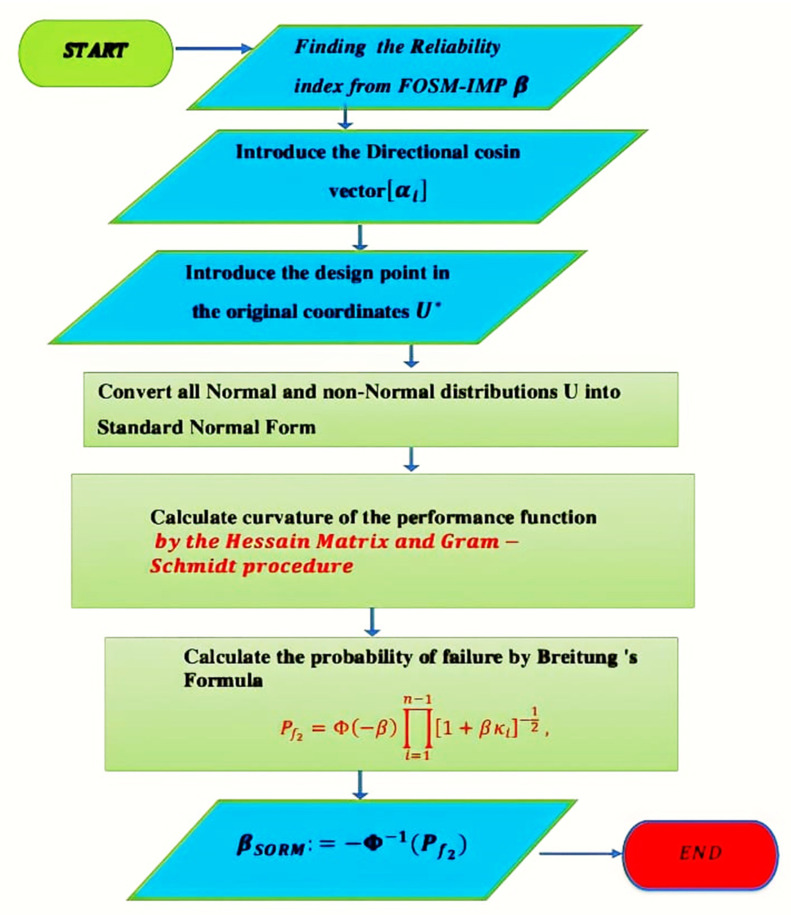
The flowchart of the SORM.

Figure 9 shows the flowchart of the SORM. To calculate the curvature $\kappa_{i}$ of the failure of a hypersurface, follow these steps:

- Construct the rotation matrix $R_{0}=\left[\begin{array}{ccc} 1 & \cdots & 0\\ \vdots & \ddots & \vdots \\ \alpha_{1} & \cdots & \alpha_{n} \end{array}\right];\;\left[\alpha_{i}\right]=\frac{{\left[\nabla G\left(\;Z^{*}\right)\right]}^{T}}{∥\nabla G\left(\;Z^{*}\right)∥}\;\mathrm{and}\;\nabla G\left(\;Z^{*}\right)=-{\left.\frac{\partial G}{\partial Z_{i}}\right|}_{\left(Z_{i}{}^{*}\right)}\;i=1‥n.$
- Using the Gram-Schmidt orthogonalization procedure, convert the matrix $R_{0}$ to the orthonormal matrix *R*.
- Transform the vector $\left(Z_{i}{}^{*}\right)$ to anther vector denoted by $\left(Y_{i}\right)$ by the relationship $Y_{i}=R\;Z_{i}{}^{*}$.
- Construct the Hessian matrix (*D*) containing the second derivative of a limit stat at the design point in the standard normal Space, where $D=\left[\left[\nabla^{2}G\left(\;Z^{*}\right)\right]={\left.\left[\frac{\partial 2G\left(.\right)}{\partial Z_{i}\partial Z_{j}}\right]\right|}_{Z^{*}}i=1‥n,\;j=1‥n\;\right]$.
  Compute matrix *A*, whose elements $a_{ij}$ are calculated as $\;A={\left[a_{ij}\right]}_{n}=\left[\frac{RDR^{T}}{\sqrt{∥\nabla G\left(\;Z^{*}\right)∥}}\right]$. The last column and last row in the *A* matrix and the last row in vector $U_{i}$ are dropped because they present $\nabla G\left(\;Z^{*}\right)$, which is the first derivative of the Taylor series that exists in the calculation of β by FOSM(RF-IMP).
- Finally, the $\kappa_{i}$ are determined as the eigenvalues of matrix *A* of size (*n* − 1) * (*n* − 1).
- After calculating the $\kappa_{i}$ using Breitung’s formula to calculate the probability of failure and Equation (20) to evaluate the reliability index.

## 5. Analysis

### 5.1. Comparison between Reliability Methods

**Figure 10:**
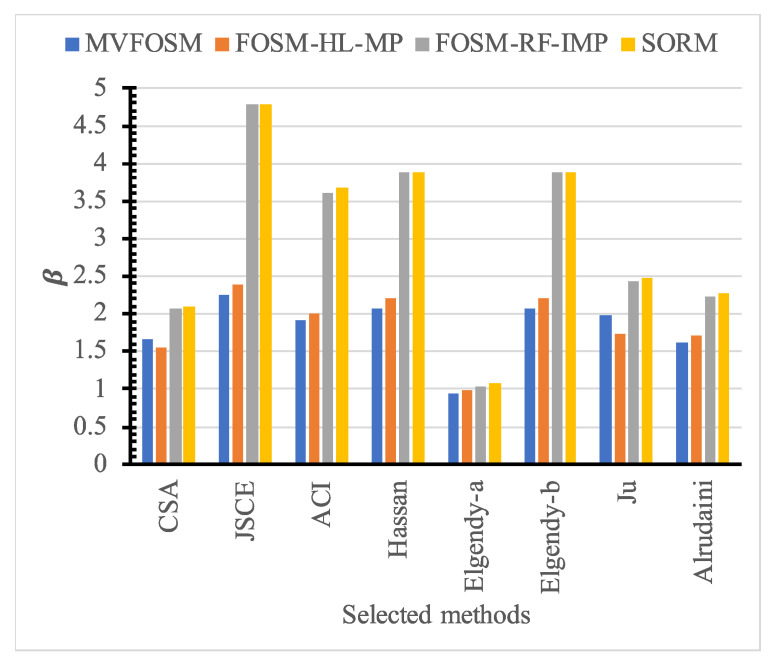
Reliability index for selected models using different reliability methods at the specific design point.

The evaluation of the reliability analysis methods was conducted using one single design vector of an FRP-reinforced concrete slab, where *E* = 80,000 MPa, *d* = 150 mm, $f_{c}^{\prime }$ = 30 MPa, ρ = 1%, and b=C = 100 mm. Table 5 and Figure 10 show the reliability index calculated for the single design vector using the various reliability analysis methods. The reliability indices predicted using the accurate SORM are quite like those predicted by the FOSM-RF-IMP, while those predicted by the MVFOSM and the FOSM-HL-MP are quite different. Thus, from this point forward, the FOSM-RF-IMP is being implemented.

### 5.2. Evaluation of the Selected Model

**Figure 11:**
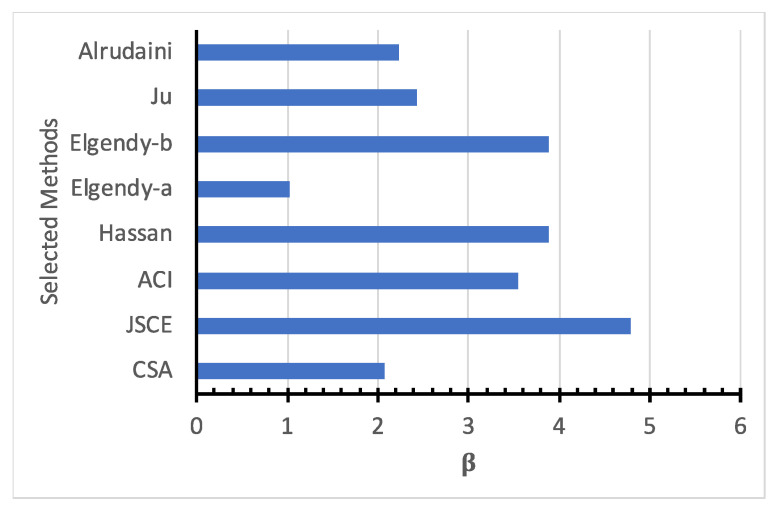
Reliability index for selected models at the specific design point.

**Figure 12:**
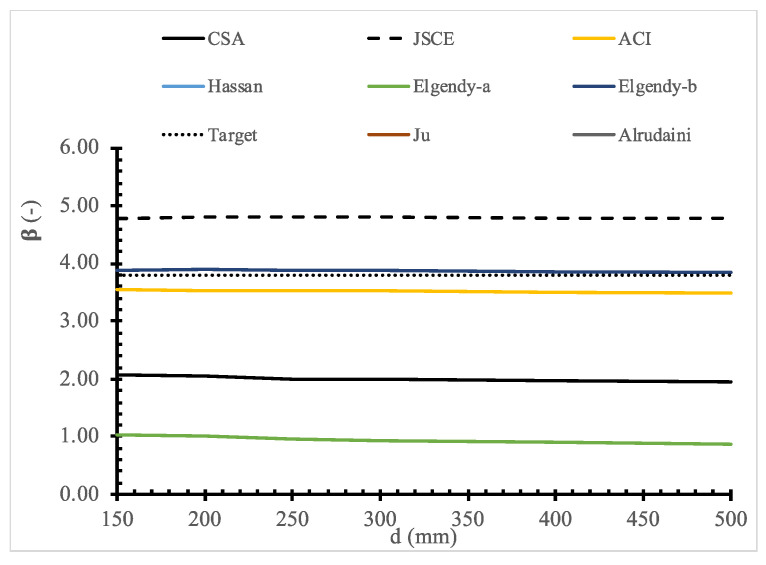
Reliability index for selected models versus *d*.

**Figure 13:**
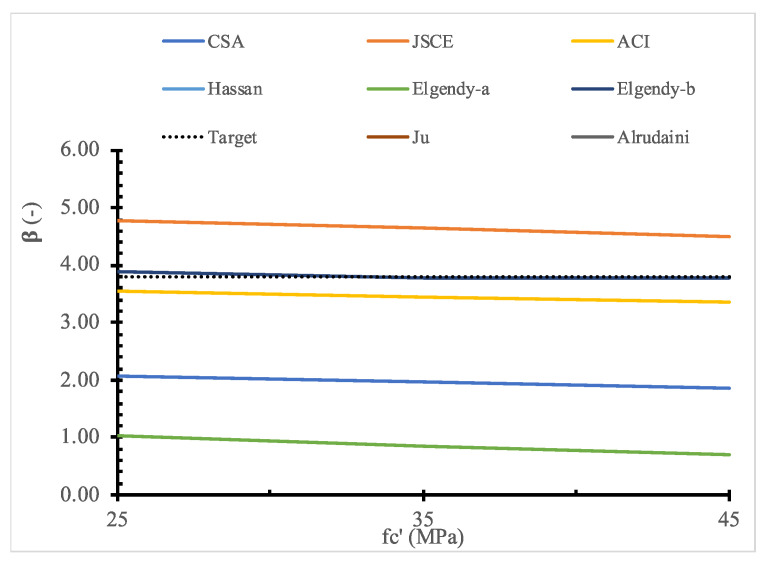
Reliability index for selected models versus $f_{c}^{\prime }$.

**Figure 14:**
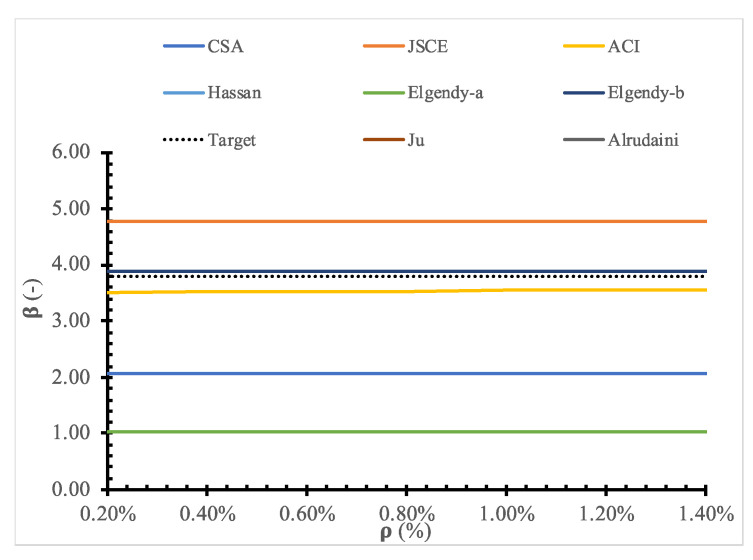
Reliability index for selected models versus *ρ* (%).

**Figure 15:**
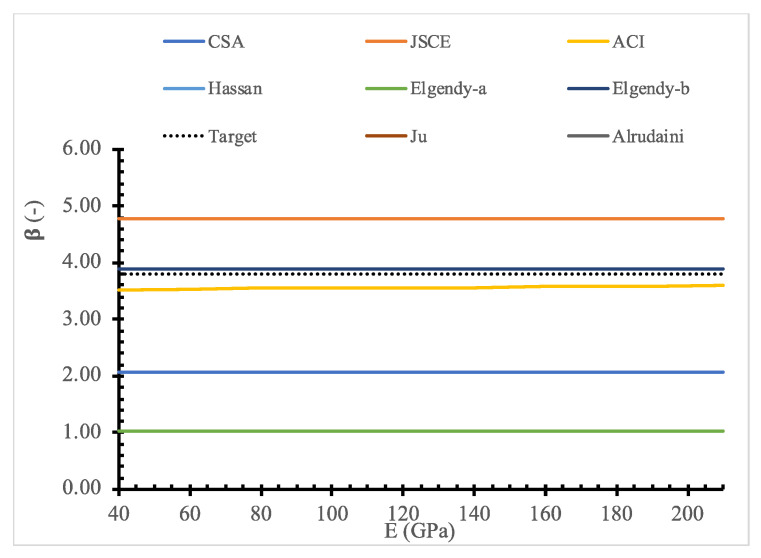
Reliability index for selected models versus *E*.

**Figure 16:**
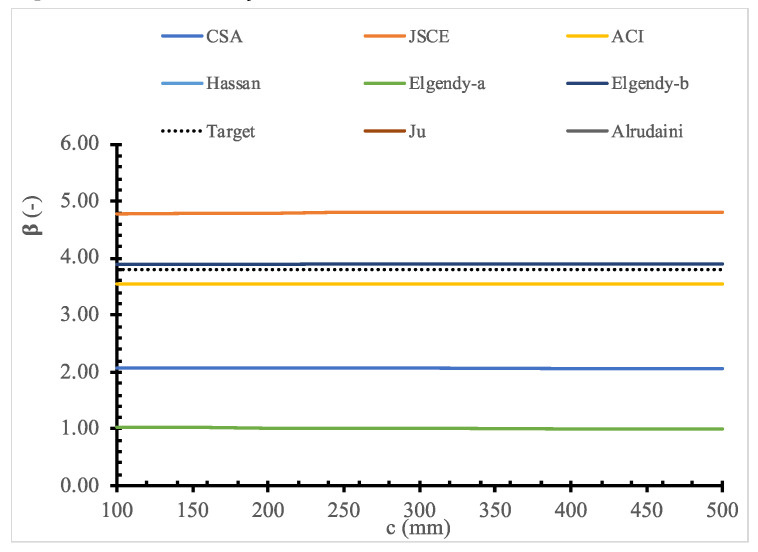
Reliability index for selected models versus *c*.

**Figure 17:**
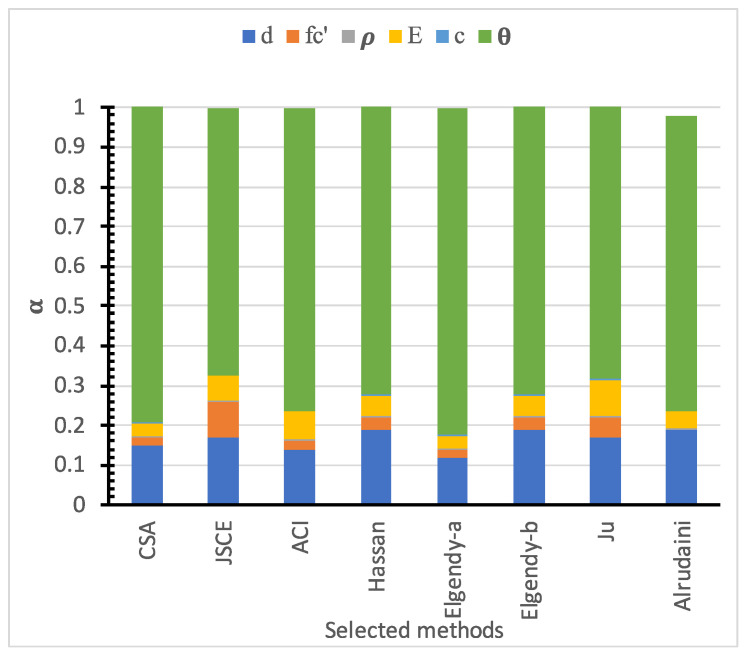
The sensitivity index for basic variables using all selected models.

To evaluate the existing design models, a parametric study for the full range of values for each parameter is shown in Figure 11, Figure 12, Figure 13, Figure 14, Figure 15, Figure 16 and Figure 17 and Table 6, Table 7, Table 8, Table 9, Table 10 and Table 11. The FOSM-RF-IM was implemented because it provides the same accuracy as the SORM while being much simpler.

#### 5.2.1. Reliability Indices

For the various design models shown in Table 1, the reliability index is calculated for the single vector as shown in Table 6 and Figure 11. There was a significant difference in the reliability index value for different design codes and models. The JSCE is the most reliable design code, followed by the ACI and the CSA, while Elgendy-b and Hassan are the most reliable design models, followed by Ju and Alrudainin. On the other hand, Elgendy-a is the least reliable. However, not all the models had a reliability index higher than the target value of 3.95 [31]. Thus, further investigation is needed into the reliability of current models.

Moreover, the effect of each parameter on the reliability is evaluated, where the reliability index is calculated for one variable at a time. At the same time, the values of other variables are kept constant. For example, Table 7, Table 8, Table 9, Table 10 and Table 11 show that the reliability of the selected models drops down with the increase in the slab size and concrete compressive strength. In contrast, the reliability of all design models is not significantly affected by variation in Young’s modulus, loading area dimensions, and flexure reinforcement ratio.

#### 5.2.2. Sensitivity Analysis

For the various design models shown in Table 1, the sensitivity factors concerning the variables *d*, $f_{c}^{\prime }$, ρ, and *E*, *c*, are calculated for the single vector as shown in Figure 17 and Table 6. There was a significant difference in the sensitivity factor values for different models. The model sensitivity is the most influential parameter, followed by slab size, concrete compressive strength, Young’s modulus, and, finally, the flexure reinforcement ratio and loading area dimensions. Moreover, the effect of each parameter on the reliability was evaluated, where the sensitivity factors are calculated for one variable at a time while the values of other variables are kept constant. Table 7, Table 8, Table 9, Table 10 and Table 11 show the sensitivity factors of the slab size drops down with the increase in the slab size, similar to the concrete compressive strength and Young’s modulus. In contrast, variation in loading area dimensions and the flexure reinforcement ratio does not significantly affect the sensitivity factors.

## 6. Conclusions

A state-of-the-art reliability analysis method was implemented for the punching shear design of FRP-reinforced concrete slabs. Several design models were examined, where the reliability index and sensitivity factor were calculated for each model. The following conclusions were reached:

- The first-order second moment method with the Rackwitez–Fissler improved matrix procedure is more accurate than the mean-value first-order second moment and the Hasofer and Lindt matrix procedure, and as precise as the second-order reliability method.
- Many of the existing models lack the required reliability needed for a safe design. Thus, further investigation in this area is required, emphasizing reliability.
- The importance of effective variables was identified as follows: (1) model accuracy; (2) slab effective depth; (3) concrete compressive strength; (4) Young’s modulus; and (5) flexure reinforcement ratio and loading area dimensions.

## Figures and Tables

**Table 1:** Experimental database for FRP-reinforced concrete slabs under punching shear.

Reference	*N*	*A*	*B*	*b*	*c*	*d*	$f_{c}^{\prime }$	ρ	*FRP*	*E*	*V*
		(mm)	(mm)	(mm)	(mm)	(mm)	(MPa)	(%)	Type *	(GPa)	(kN)
[51]	4	690	690	75–100	75	61	36–45	0.95	CFRP	113	78–99
[52]	3	600	600	100	100	55	41–53	0.31	CFRP	100	61–72
[53]	6	1800	1500	250	250	76	30	1.49–2.05	CFRP	143–156	179–201
[54]	12	3000	1800	575	225	175	43–55	1	GFRP and CFRP	39–160	500–1183
[55]	13	1000	1000	80–230	80–230	95–126	32–118	0.19–1.22	CFRP and GFRP	37–149	142–347
[56]	5	2000	2500	250	150	162	42	0.28	GFRP	85	534–698
[57]	3	1800	3000	575	225	165	59	0.57	CFRP	147	1000–1328
[58]	1	2000	4000	500	250	138	35	2.4	GFRP	42	756
[59]	5	2000	2000	200	200	142	29–47	0.18–0.47	GFRP and CFRP	45–110	170–317
[60]	3	2150	2150	250	250	120	29.5–37.5	0.73–1.46	GFRP	28–34	206–260
[61]	1	1760	1760	250	250	75	45	1	CFRP and GFRP	100	234
[62]	4	1830	1830	250	250	100	26–40	1.05–1.67	GFRP	42	210–249
[63]	5	2000–2300	2000	635	250	175	27.6	0.95–0.98	GFRP	33	537–897
[64]	5	3000	2500	600	250	159	44–49.6	0.35–1.99	GFRP and CFRP	38–122	674–799
[65]	2	1830	1830	250	250	100	35–71	1.05–1.18	GFRP	42	218–275
[66]	7	1900	1900	250	250	100	25–98	0.36–0.75	CFRP	120	251–446
[67]	6	1900	1900	250	250	110-160	62-70	1.0–1.5	GFRP	43.5	282–589
[68]	7	1760	1000	250	250	120	25	0.94–1.48	CFRP	100	97–211
[69]	2	3000	2500	600	250	156	44.1	1.2	GFRP	44.5	707–735
[70]	4	1200	1200	200	200	82–112	33–40	0.81–1.54	GFRP	46	165–230
[71]	4	2300	2300	225	225	110	36.3	1.17–3	GFRP	48.2	222–330
[72]	7	1500	1500	150	150	135	22–42	0.29–0.55	BFRP	100	145–275
[73]	7	300	300	25	25	45	47.8–179	0.78	GFRP and CFRP	76–230	39–98
[74]	7	3000	2500	600	250	110–155	35–65	0.70–1.20	GFRP	43	362–732
[75]	5	1500	1500	150	150	130	22–45	0.29–0.55	GFRP	45.6	167–252
[76]	3	2200	2200	200	200	130	48.8	0.48–0.92	GFRP	48	180
[77]	19	2500	2500	300	300	131–284	32–75	0.30–1.61	GFRP	48–57	329–1248
[78]	6	2800	1500	300	300	160	41	0.85–1.70	GFRP	60.5	159–277
[79]	4	1425	500	500	25	117–119	65–69	0.6	GFRP	54–67.4	295–365
[80]	3	2600	1450	300	300	160	80–85	0.87–1.70	GFRP	60.5–69.3	251–288
[81]	6	3000	2000	600	250	160	42–48	0.40–1.20	BFRP	69.3	436–716
[24]	4	2600	2600	300	300	160	38–70	0.65–1.30	GFRP	65–69	363–719
[82]	1	800	800	250	250	176	59	0.7	GFRP	68	719
[83]	3	2800	2800	300	300	160	80–87	0.98–1.93	GFRP	65	461–604
[84]	8	600	600	100	100	80	46–60	0.3–0.90	CFRP	144	57–129
[85]	1	1600	1600	200	200	125	24.97	0.89	CFRP	123	262
Mean		1961	1736	301	212	131	46	0.94		80	416
Minimum		300	300	25	25	45	22	0.18		28	39
Maximum		3000	4000	635	300	284	179	3.76		230	1600

* CFRP is carbon FRP, and GFRP is glass FRP. *N* is number of tested specimens, punching shear failure load (V), Young’s modulus (E), effective depth (d), concrete compressive strength ($f_{c}^{\prime }$), flexure reinforcement ratio (ρ), column dimensions (b, c), and slab dimensions (A, B).

**Table 2:** Selected models.

Reference	Punching Shear Formula	Symbols
JSCE [86]	$V=\beta_{d}\beta_{\rho }\beta_{r}f_{Pcd}b_{0.5d}d$	βd=1000d14≤1.5, βρ=100ρEEs13≤1.5, $\beta_{r}=1+\frac{1}{\left(1+\frac{0.25b_{0.5d}}{d}\right)},\;$ $f_{Pcd}=0.2\sqrt{f_{c}^{\prime }}\;\leq 1.2$ MPa, $b_{0.5d}=4\left(c+d\right)$
CSA [23]	V=b0.5dd0.0281+2βcEρfc′130.147Eρfc′130.19+αsdb0.5d0.056Eρfc′13	$\beta_{c}=$$\;1,\;\alpha_{s}$ = 4, 3, 4, 3, 3 for an inner, edge, corner connection. $b_{0.5d}=4\left(c+d\right)$
ACI [7]	$V=0.8\sqrt{f_{c}^{\prime }}kdb_{0.5d}$	$k=\sqrt{2\rho n+{\left(\rho n\right)}^{2}}-\rho n$$,\;n=\frac{E}{E_{c}}$ $E_{c}=4750\sqrt{f_{c}^{\prime }}$*,* $b_{0.5d}=4\left(c+d\right)$
Hassan [77]	V=0.0650.65+4db0.5dEρfc′13125d16b0.5dd	$b_{0.5d}=4\left(c+d\right)$
Elgendy-a [6]	V=0.33fc′120.62Eρ131+2αsdb0.5d1.2Nb0.5dd	$\alpha_{s}$ = 4, 3, 4, 3, 3 for an inner, edge, corner connection. $\;N=1.\;b_{0.5d}=4\left(c+d\right)$
Elgendy-b [6]	V=0.0650.65+αsdb0.5dEρfc′13125d16b0.5dd	$\alpha_{s}$ = 4, 3, 4, 3, 3 for an inner, edge, corner connection. $\;b_{0.5d}=4\left(c+d\right)$
Ju [87]	V=2.3 100ρEEsfc′12db0.5d12b0.5dd	$b_{0.5d}=4\left(c+d\right)$
Alrudaini [88]	V=0.41 Eρfc′13db015b0d	$b_{0}=4\left(c+2d\right)$

**Table 3:** Selected models’ variability.

Reference	Normal Distribution		Log-Normal Corrected	
	$\theta_{mean}$	$\theta_{c.o.v}$	$\theta_{mean}$	$\theta_{c.o.v}$
JSCE	2.87	0.36	2.87	0.35
CSA	1.20	0.37	1.20	0.38
ACI	2.20	0.39	2.20	0.38
Hassan	2.19	0.32	2.20	0.35
Elgendy-a	0.72	0.31	0.73	0.36
Elgendy-b	2.19	0.32	2.20	0.35
Ju	1.24	0.32	1.25	0.36
Alrudaini	1.12	0.32	1.12	0.32

**Table 4:** Variability of basic variables based on JCSS probabilistic model code.

Variable	Distribution	Nominal Value	Mean Value	Standard Deviation	Coefficient of Variation
Concrete compressive strength ($f_{c}^{\prime }$, MPa)	Log-normal	25	38.8 *	4.67 *	-
		35	47.2 *	4.26 *	-
		45	53.6 *	3.76 *	-
Effective depth (d, mm)	Normal	$d_{n}$	$d_{n}+10$	10	-
Column dimension (c, mm)	Normal	$c_{n}$	$1.003c_{n}$	4 + 0.006$c_{n}$	-
Flexure reinforcement ratio (ρ, %)	Normal	$\rho_{n}$	$\rho_{n}$	-	0.02
Young’s modulus (E, MPa)	Normal	$E_{n}$	$E_{n}$	-	0.15
Model uncertainty (θ)	Log-normal	-	See Table 3		

* Corrected values to include the log-normal distribution.

**Table 5:** Comparison between reliability index (β) using different methods.

Method	CSA	JSCE	ACI	Hassan	Elgendy-a	Elgendy-b	Ju	Alrudaini
MVFOSM	1.66	2.26	1.91	2.08	0.95	2.08	1.98	1.61
FOSM-HL-MP	1.54	2.39	2.01	2.20	0.99	2.20	1.73	1.71
FOSM-RF-IMP	2.07	4.78	3.60	3.89	1.03	3.89	2.43	2.23
SORM	2.10	4.78	3.68	3.89	1.07	3.89	2.47	2.27

**Table 6:** Reliability and sensitivity index for all selected models.

Method	CSA	JSCE	ACI	Hassan	Elgendy-a	Elgendy-b	Ju	Alrudaini
β	2.07	4.78	3.55	3.89	1.03	3.89	2.43	2.23
$\alpha_{d}^{\;2}$	15%	17%	14%	19%	12%	19%	17%	19%
$\alpha_{f}^{\;2}$	2%	9%	2%	3%	2%	3%	5%	0%
$\alpha_{\rho }^{\;2}$	0.3%	0.4%	0.4%	0.4%	0.4%	0.4%	0.4%	0.4%
$\alpha_{E}^{\;2}$	0.03	6%	7%	5%	3%	5%	9%	4%
$\alpha_{c}^{\;2}$	0.4%	0.3%	0.3%	0.3%	0.3%	0.3%	0.3%	0.3%
$\alpha_{\theta }^{\;2}$	80%	67%	76%	73%	82%	73%	69%	74%

**Table 7:** Effect of size on selected models’ reliability.

*d*	CSA		JSCE		ACI		Hassan		Elgendy-a		Elgendy-b		Ju		Alrudaini	
(mm)	β	$\alpha_{d}^{\;2}$	β	$\alpha_{d}^{\;2}$	β	$\alpha_{d}^{\;2}$	β	$\alpha_{d}^{\;2}$	β	$\alpha_{d}^{\;2}$	β	$\alpha_{d}^{\;2}$	β	$\alpha_{d}^{\;2}$	β	$\alpha_{d}^{\;2}$
150	2.07	15%	4.78	17%	3.55	14%	3.89	19%	1.03	12%	3.89	19%	2.43	17%	2.23	19%
200	2.04	8%	4.81	8%	3.54	6%	3.9	9%	1	8%	3.9	9%	2.41	9%	2.2	10%
250	2.01	5%	4.81	5%	3.53	5%	3.89	6%	0.96	5%	3.89	6%	2.38	6%	2.18	7%
300	1.99	3%	4.81	4%	3.52	3%	3.88	4%	0.93	3%	3.88	4%	2.37	4%	2.16	5%
400	1.96	2%	4.8	2%	3.5	2%	3.87	2%	0.9	2%	3.87	2%	2.34	2%	2.13	3%
500	1.95	1%	4.79	1%	3.49	1%	3.85	1%	0.87	1%	3.85	1%	2.32	1%	2.11	2%

**Table 8:** Effect of concrete compressive strength on selected models’ reliability.

$f_{c}^{\prime }$	CSA		JSCE		ACI		Hassan		Elgendy-a		Elgendy-b		Ju		Alrudaini	
(MPa)	β	$\alpha_{f\prime }^{\;2}$	β	$\alpha_{f\prime }^{\;2}$	β	$\alpha_{f\prime }^{\;2}$	β	$\alpha_{f\prime }^{\;2}$	β	$\alpha_{f\prime }^{\;2}$	β	$\alpha_{f\prime }^{\;2}$	β	$\alpha_{f\prime }^{\;2}$	β	$\alpha_{f\prime }^{\;2}$
25	2.07	15%	4.78	9%	3.55	2%	3.89	3%	1.03	3%	3.89	3%	2.43	5%	2.23	0.4%
35	1.96	1%	4.65	4%	3.45	1%	3.78	1%	0.86	2%	3.78	1%	2.27	2%	2.1	0.4%
45	1.86	0%	4.5	2%	3.36	0%	3.78	1%	0.7	1%	3.78	1%	2.11	1%	1.99	0.3%

**Table 9:** Effect of flexure reinforcements on selected models’ reliability.

*ρ*	CSA		JSCE)		ACI		Hassan		Elgendy-a		Elgendy-b		Ju		Alrudaini	
(%)	β	$\alpha_{\rho }^{\;2}$	β	$\alpha_{\rho }^{\;2}$	β	$\alpha_{\rho }^{\;2}$	β	$\alpha_{\rho }^{\;2}$	β	$\alpha_{\rho }^{\;2}$	β	$\alpha_{\rho }^{\;2}$	β	$\alpha_{\rho }^{\;2}$	β	$\alpha_{\rho }^{\;2}$
0.2%	2.07	0.4%	4.78	0.4%	3.51	0.4%	3.89	0.4%	1.03	0.4%	3.89	0.4%	2.43	0.4%	2.23	0.4%
0.4%	2.07	0.4%	4.78	0.4%	3.52	0.4%	3.89	0.4%	1.03	0.4%	3.89	0.4%	2.43	0.4%	2.23	0.4%
0.6%	2.07	0.4%	4.78	0.4%	3.53	0.4%	3.89	0.4%	1.03	0.4%	3.89	0.4%	2.43	0.4%	2.23	0.4%
0.8%	2.07	0.4%	4.78	0.4%	3.54	0.4%	3.89	0.4%	1.03	0.4%	3.89	0.4%	2.43	0.4%	2.23	0.4%
1%	2.07	0.4%	4.78	0.4%	3.55	0.4%	3.89	0.4%	1.03	0.4%	3.89	0.4%	2.43	0.4%	2.23	0.4%
1.4%	2.07	0.4%	4.78	0.4%	3.56	0.4%	3.89	0.4%	1.03	0.4%	3.89	0.4%	2.43	0.4%	2.23	0.4%
1.6%	2.07	0.4%	4.78	0.4%	3.57	0.4%	3.89	0.4%	1.03	0.4%	3.89	0.4%	2.43	0.4%	2.23	0.4%
2%	2.07	0.4%	4.78	0.4%	3.58	0.4%	3.89	0.4%	1.03	0.4%	3.89	0.4%	2.43	0.4%	2.23	0.4%

**Table 10:** Effect of Young’s modulus on selected models’ reliability.

*E*	CSA		JSCE		ACI		Hassan		Elgendy-a		Elgendy-b		Ju		Alrudaini	
(GPa)	β	$\alpha_{E}^{\;2}$	β	$\alpha_{E}^{\;2}$	β	$\alpha_{E}^{\;2}$	β	$\alpha_{E}^{\;2}$	β	$\alpha_{E}^{\;2}$	β	$\alpha_{E}^{\;2}$	β	$\alpha_{E}^{\;2}$	β	$\alpha_{E}^{\;2}$
40	2.07	4%	4.78	6%	3.52	8%	3.89	5%	1.03	2%	3.89	5%	2.43	9%	2.23	4%
60	2.07	4%	4.78	6%	3.54	8%	3.89	5%	1.03	2%	3.89	5%	2.43	9%	2.23	4%
80	2.07	4%	4.78	6%	3.55	7%	3.89	5%	1.03	2%	3.89	5%	2.43	9%	2.23	4%
100	2.07	4%	4.78	6%	3.55	7%	3.89	5%	1.03	2%	3.89	5%	2.43	9%	2.23	4%
120	2.07	4%	4.78	6%	3.56	6%	3.89	5%	1.03	2%	3.89	5%	2.43	9%	2.23	4%
140	2.07	4%	4.78	6%	3.57	6%	3.89	5%	1.03	2%	3.89	5%	2.43	9%	2.23	4%
160	2.07	3%	4.78	6%	3.58	6%	3.89	5%	1.03	2%	3.89	5%	2.43	9%	2.23	4%
190	2.07	3%	4.78	6%	3.59	5%	3.89	5%	1.03	2%	3.89	5%	2.43	9%	2.23	4%
210	2.07	3%	4.78	6%	3.6	5%	3.89	5%	1.03	2%	3.89	5%	2.43	9%	2.23	4%

**Table 11:** Effect of loading area dimension on reliability and sensitivity indices.

*c*	CSA		JSCE		ACI		Hassan		Elgendy-a		Elgendy-b		Ju		Alrudaini	
(mm)	β	$\alpha_{c}^{\;2}$	β	$\alpha_{c}^{\;2}$	β	$\alpha_{c}^{\;2}$	β	$\alpha_{c}^{\;2}$	β	$\alpha_{c}^{\;2}$	β	$\alpha_{c}^{\;2}$	β	$\alpha_{c}^{\;2}$	β	$\alpha_{c}^{\;2}$
100	2.07	0.3%	4.78	0.3%	3.55	0.3%	3.89	0.3%	1.03	0.3%	3.89	0.3%	2.43	0.3%	2.23	0.3%
150	2.07	0.3%	4.8	0.3%	3.55	0.3%	3.89	0.3%	1.03	0.3%	3.89	0.3%	2.43	0.3%	2.23	0.3%
200	2.07	0.3%	4.8	0.3%	3.55	0.3%	3.89	0.3%	1.02	0.3%	3.89	0.3%	2.43	0.3%	2.23	0.3%
250	2.07	0.3%	4.81	0.3%	3.55	0.3%	3.9	0.3%	1.02	0.3%	3.9	0.3%	2.43	0.3%	2.22	0.3%
300	2.07	0.3%	4.81	0.3%	3.55	0.3%	3.9	0.3%	1.01	0.3%	3.9	0.3%	2.43	0.3%	2.22	0.3%
400	2.06	0.3%	4.81	0.3%	3.55	0.3%	3.9	0.3%	1.00	0.3%	3.9	0.3%	2.43	0.3%	2.22	0.3%
500	2.06	0.3%	4.81	0.3%	3.55	0.3%	3.9	0.3%	1.00	0.3%	3.9	0.3%	2.43	0.3%	2.22	0.3%

## Data Availability

All data are available within the manuscript.
